# Evaluation of strategies for the prevention of mother-to-child transmission of HIV in Cameroon: a mixed qualitative and quantitative cross-sectional analysis from the Adamawa region of Cameroon

**DOI:** 10.1186/s12889-022-14871-6

**Published:** 2022-12-31

**Authors:** Bibiane Siaheu Kameni, Jobert Richie Nansseu, Jean Joel Bigna, Sandra Ayuk Tatah, Catherine Seyler

**Affiliations:** 1 Adamawa Regional Technical Group for the Fight Against HIV/AIDS and Regional Delegation of Public Health, National AIDS Control Program of the Ministry of Public Health, Ngaoundéré, Cameroon; 2 Department for the Control of Disease, Epidemics and Pandemics of the Ministry of Public Health, Yaoundé, Cameroon; 3 Department of Public Health, Faculty of Medicine and Biomedical Sciences of the University of Yaoundé I, Yaoundé, Cameroon; 4 Department of Epidemiology and Public Health, Centre Pasteur of Cameroon, Rue Henry Dunant; P.O, 1274 Yaoundé, Cameroon; 5 Department of Paediatrics and Specialties, Faculty of Medicine and Biomedical Sciences of the University of Yaoundé I, Yaoundé, Cameroon; 6 Department of Medical Information, APHM, Marseille, France

**Keywords:** Prevention of mother-to-child transmission, HIV, Cameroon, Sub-Saharan Africa

## Abstract

**Background:**

To accelerate the fight against HIV/AIDS and eliminate the mother-to-child transmission (MTCT) of the virus, Cameroon has implemented and intensified several strategies despite which numerous children continue to be born infected with HIV. This study aimed to evaluate these strategies put in place for the prevention of MTCT (PMTCT) in Cameroon.

**Methods:**

A qualitative and quantitative cross-sectional analysis was conducted in seven PMTCT care units situated in the Adamawa region of the country. The qualitative analysis included 16 individual interviews of key informants and observations of attitudes and practices being implemented in each unit. On the other hand, the quantitative analysis targeted 106 known HIV-positive breastfeeding women being followed-up at the unit.

**Results:**

Task-shifting and sharing was effective, but majority of staffs had not received any specific training on PMTCT. Moreover, the integration of PMTCT within the maternal, neonatal and child health services remained ineffective, especially in health facilities of heavy workload. The coordination of PMTCT services was led by a well-designated focal person; however, his/her roles and responsibilities had not clearly been defined. Of the 106 women enrolled, 59.4% had a level of knowledge on PMTCT less than 80%. Similarly, their attitudes and practices towards PMTCT were inadequate or inaccurate in more than 60% of cases.

**Conclusion:**

PMTCT strategies are globally well known and accepted by healthcare professionals. However, weaknesses have been figured out regarding service integration, task shifting and sharing, and coordination. In addition, beneficiaries’ attitudes and practices are insufficient, and their level of knowledge does not guarantee to lessen the risk of MTCT of HIV.

**Supplementary Information:**

The online version contains supplementary material available at 10.1186/s12889-022-14871-6.

## Background

Discovered in the 1980s, the human immunodeficiency virus (HIV) infection is one of the largest pandemics the world has ever witnessed [1]. According to the latest UNAIDS report, in 2020, over 37.7 million people were living with HIV worldwide, 4.7 million of them in West and Central Africa. According to the same report, every week, about 6,000 young women aged between 15 and 24 years become infected with HIV, worldwide [2]. In sub-Saharan Africa in particular, 3 out of 4 new infections among women occur between ages 15 to 19; on another hand, women aged 15 to 24 are twice as infected as men in the same age group. The burden of HIV is much higher in women than in men, globally [2]. There are several routes of HIV transmission including the mother-to-child transmission (MTCT) which may occur during pregnancy, childbirth, or breastfeeding. MTCT is the main route of transmission in children aged less than 15 years. In 2020, there were around 131,000 new HIV infections among children aged 0 to 14, about 40% of which occurred in Central and West Africa [2]. To curb the burden of HIV in children, the global targets were to accelerate HIV response and eliminate MTCT of HIV by reducing the number of newly infected children to less than 2,000 per year, by 2020 [3].

Among West and Central African countries, Cameroon ranks at second with the highest burden of HIV, after Nigeria [4]. According to the latest Demographic and Health Survey (DHS), the national prevalence of HIV is at 2.7%: 3.4% in women and 1.9% in men [5]. In response, the country has developed a National Strategic Plan for HIV/AIDS and Sexually Transmissible Infections which major objectives included the reduction of new infections among children, adolescents and adults, elimination of MTCT of HIV, and reduction of HIV-associated mortality [6].

Accordingly, Cameroon has defined numerous strategies and priority interventions including the prevention of MTCT (PMTCT) of HIV extensively described in its National Guidelines released in 2015 and later revised in 2019 [7, 8]. PMTCT interventions aimed to reinforce community participation for elimination of MTCT, promote continual utilization of maternal, neonatal and infant health services, offer free HIV testing to all pregnant women, offer free dispensation of antiretroviral therapy (ART) to HIV positive pregnant and breastfeeding women and assure retention on care, provide ART prophylaxis and cotrimoxazole to exposed new-borns, perform early HIV testing of exposed new-borns, and follow-up the mother–child couple until determination of the child’s final status at 18–24 months [7, 8]. In 2020 for instance, around 84.3% of health care facilities were actively delivering PMTCT interventions, nationwide [9]. But despite all these measures, many infants continue to be born infected with HIV and die early due to complications. National estimates revealed indeed that the overall HIV-positive rate among infants exposed to HIV was around 5 and 4.3%, respectively in 2017 and 2020 [10, 11]. However, to the best of our knowledge, no evaluation of the above strategies had already been carried-out in Cameroon. Results of this evaluation would describe the quality of care of HIV-infected pregnant women and identify different bottlenecks in the implementation of HIV-related PMTCT strategies. Ultimately, they would underpin the implementation of targeted strategies and actions to help improving quality of care for pregnant women and consequentially reduce MTCT of HIV in our country, and hence in other countries in the sub-region that share the same realities and challenges. In this regard, we developed and designed the present study to evaluate the different HIV-related PMTCT strategies put in place in Cameroon, as of 31 December 2018.

## Methods

### Study design and setting

**Fig. 1:**
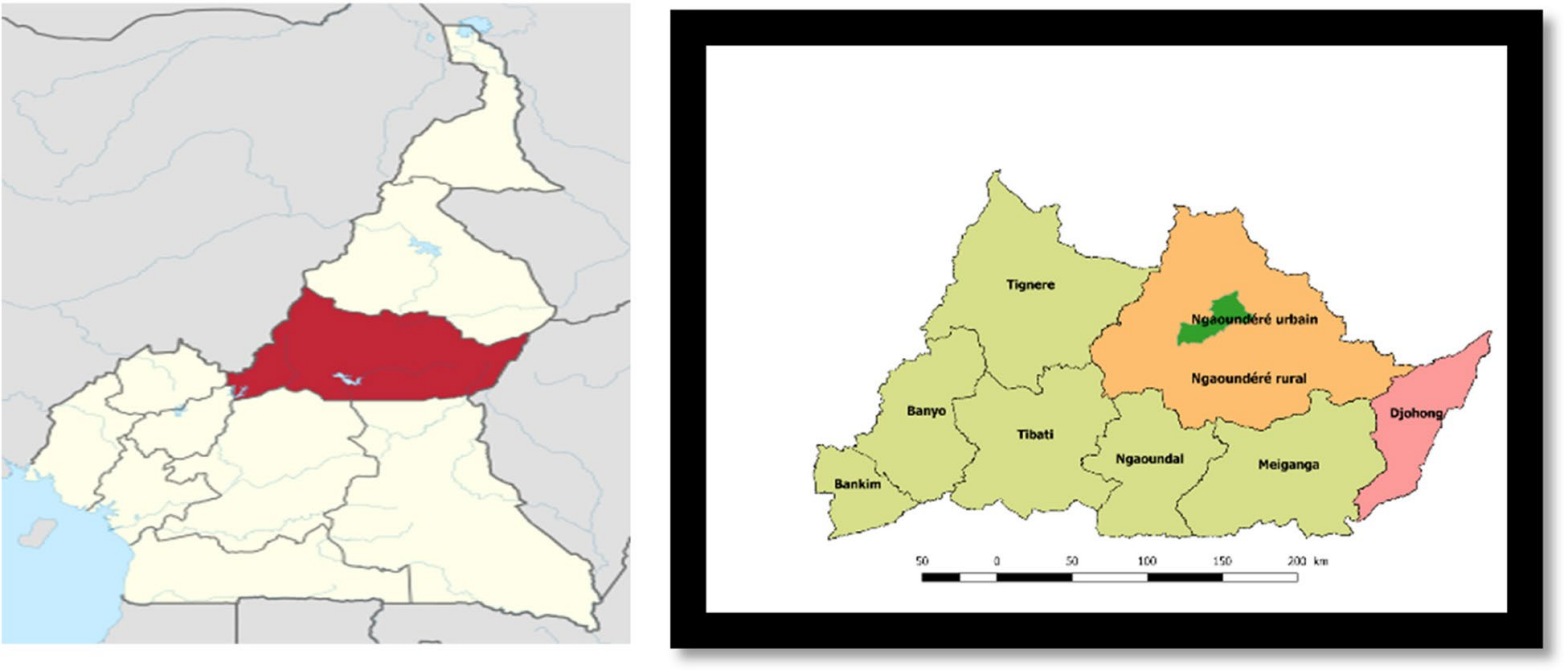
Geographic location of the Adamawa region in Cameroon (left) and repartition of its various health districts (right)

We conducted a mixed analysis combining a qualitative case study and a cross-sectional quantitative study, between January and June 2019 in seven health care facilities of the Adamawa region of Cameroon. This is one of the ten administrative regions of Cameroon, situated in the northern centre of the country, at 7° 20′ north latitude and 13° 30′ east longitude. It included nine health districts at the time of data collection (Fig. 1).

Adamawa is the third most affected region of the country, with a HIV prevalence of 4.1%: 4.7% in women and 3.4% in men [5]. Although the latest DHS did not provide data on antenatal visits desegregated by region, nearly 93.1 and 79.5% of women, respectively residing in urban and rural areas of the country had ever attended at least one antenatal visit during their last pregnancy [5]. In Adamawa, nearly 98.5% of women expected for antenatal visits attended at least one visit in 2019, and the regional prevalence of HIV was estimated at 3.3% among pregnant women [12]. Moreover, 89.9% of children aged less than 2 years had ever been breastfed in Adamawa, according to the latest DHS. Specifically, 39.7% of children aged 0–5 months had been exclusively breastfed, 64.5% of children had been breastfed until one year, and 23.2% until two years, countrywide; no regional data were available [5].

Furthermore, at the time the study was conducted, Adamawa comprised 184 health care facilities with a PMTCT coverage estimated at 94.0% (*n* = 173). However, only 71 (38.6%) health care facilities—including two with point of care—were performing dry blood smear sampling for early HIV diagnosis for exposed infants through PCR testing in national reference laboratories [12]. For the choice of the seven study sites, we conducted a purposeful sampling. First, the Adamawa Regional Hospital, which is the only regional hospital of the region with the biggest HIV treatment centre, was primarily retained. Second, we selected two health districts: one with the highest prevalence of MTCT of HIV in the region (13.3%) and the other with the lowest prevalence (0.0%), as of 31 December 2018 [13]. As a result, the Djohong and Ngaoundéré Rural health districts were retained. Further, in each of these health districts, we selected 3 health care facilities, either from the public or private sector, considering their level in the national health pyramid, i.e. a district hospital, a divisional medical centre and an integrated health care centre. The choice was based on the number/frequency of pregnant women visiting the health care facility, in other words we chose those facilities that were highly sollicitated by pregnant women. Acordingly, the Djohong District Hospital, the Ngaoui divisional medical centre, and the Kombo Laka integrated health care centre were selected in the Djohong health district, and the Mbé divisional medical centre, the Béka Hossere integrated health care centre, and the Tello islamic integrated health care centre were selected in the Ngaoundéré Rural health district.

### Study population

For the qualitative case study, we exhaustively included key healthcare professionals (physicians, nurses, nursing assistants, midwives, and psychosocial chaperones) involved in the implementation of HIV-related PMTCT strategies or in charge of HIV-infected pregnant and breastfeeding women in selected study sites. Their consent was required to participate in the study.

For the quantitative cross-sectional survey, we targeted to exhaustively recruit known HIV-infected breastfeeding women regularly followed-up in the selected facilities. This convenient choice was mainly based on the fact that these women had supposedly had many contacts with PMTCT services; hence, they were supposed to exhibit good knowledge of PMTCT interventions. Their consent was required to participate in the study. According to the regional prevalence of HIV among pregnant women, at 7.1% according to the 2011 Demographic and Health Survey [14], the minimal sample size was estimated at 102 women.

### Data collection

For the qualitative study, all targeted healthcare professionals were contacted by phone and informed of the various aspects and procedures related to the study. Once they gave their consent, an appointment was scheduled with the team of investigators at their respective workplaces. A semi-directive interview-form had previously been designed and pre-tested among non-selected participants of the same profile. On the day of appointment, the healthcare professional was met at his/her workplace and signed the informed consent form. Thereafter, a semi-directive interview was conducted using the pre-tested form. The interview was recorded with an audio Dictaphone and went through updates on different aspects of implementation of the various HIV-related PMTCT interventions in the facility, difficulties faced in implementing them, how the healthcare professional felt about what was being done, and proposed solutions for improvement. It lasted about an hour per participant. In addition, using a pre-designed observational evaluation form, health care professionals’ practices were evaluated by investigators, at different points of care delivery in the facility: antenatal consultation, delivery room, ART dispensation, and HIV out-patient consultation units. Investigators were specialists of PMTCT strategies and had no working relationships with healthcare professionals met at their workplace. During these observations, information was collected on the facility’s infrastructure, organization of task-shifting and sharing, conduct of pre- and post-test counselling, conduct of supportive sessions on compliance and therapeutic education, conduct of educational talks and support groups, collaboration between healthcare professionals, and healthcare professionals’ behaviours during delivery of care including to pregnant women. Noteworthy, at the time the study was conducted, PMTCT interventions in Cameroon were mainly focussed around care to HIV-positive pregnant and breastfeeding mothers, and to the exposed new-born until early diagnosis at six weeks [7]. The monitoring and follow-up of cohorts of exposed infants from six weeks to final diagnosis at 18–24 months was not yet clearly captured nationwide, until the National Guidelines for the prevention and management of HIV were revised in 2019 [8], and the intervention implemented as from late 2020-early 2021. Supplementary Table 1 presents the standard package of facility-based PMTCT care pre-, per-, and post-partum, and to the exposed infant according to the latest national consolidated guidelines [8]. For the cross-sectional quantitative study, women were met at the study site on their following-up appointment day. Using a pre-conceived and pre-tested questionnaire, information was collected on sociodemographic background, clinical and biological parameters, therapeutic follow-up during pregnancy, and during and after delivery. In addition, the questionnaire assessed the participant’s knowledge regarding PMTCT of HIV. Of note, data on viral load were not available, considering that at the time the study was conducted, viral load testing was not yet performed in any of the health care facilities of the Adamawa region of Cameroon.

### Data synthesis and analysis

Qualitative data were transcribed and analysed thematically, inductively, and deductively. Information from individual interviews and observations was triangulated for validity of results. From these analyses were derived the description of HIV-infected pregnant women care as well as strengths, weaknesses, opportunities, and threats of implementing HIV-related PMTCT strategies, highlighting different bottlenecks and ways of improvement.

Quantitative data were entered and coded using Microsoft Excel software, and subsequently analysed with R version 3.3.1 (The R Foundation for statistical computing, Vienna, Austria). Results are presented in the form of frequencies (percentages) for qualitative variables, and median (interquartile range, IQR) for quantitative variables.

### Ethical considerations

Before commencing the study, an ethical clearance was granted by the Regional Ethical Review Committee of the Adamawa Regional Delegation of the Ministry of Public Health. Additionally, administrative authorizations were obtained from the Regional Delegation, selected health districts, and health care facilities. All aspects and procedures related to the study were presented and explained to each potential participant. We subsequently included only those who voluntarily agreed to be part of the study. Accordingly, they signed an informed consent form. All information collected during this study was completely anonymous and kept confidential. All meta-data collected during the study were destroyed after completion of data analysis. The study was performed in accordance with the Declaration of Helsinki and all participants gave informed consent.

## Results

### Results of qualitative analysis

A total of 16 individual interviews were conducted, involving five nurses, four midwives, four psychosocial chaperones, and three nursing assistants. All health care facilities irrespective of their level in the health pyramid had a PMTCT unit comprising an antenatal consultation care service, a maternity ward, and an ART monitoring and dispensation unit for the follow-up of patients living with HIV including pregnant and breastfeeding women. Supplementary Table 2 summarizes the strengths, weaknesses, opportunities, and threats derived at the end of this analysis.

#### Organization of services, provision of care, and compliance with management guidelines

Concerning integration of services, it appeared that divisional medical centres and integrated health care centres were satisfactorily integrating PMTCT with maternal, neonatal and child health services. Indeed, PMTCT interventions, antenatal, per- and post-partum consultations were delivered in the same unit/compound, as well as provision of HIV-testing and ART dispensation. As such, a mother who comes for postnatal consultation, in addition to receiving her ART, is also offered a follow-up consultation for her baby in the same room, including administration of prophylactic ART for the new-born, early HIV diagnosis, administration of cotrimoxazole, and in some cases vaccination. However, at regional and district hospital levels, some PMTCT interventions were delivered far from maternal, neonatal and child health services such as ART intitation, follow-up appointments for ART dispensation to HIV-positive mothers, and follow-up of the exposed newborn including early HIV diagnosis.

Regarding task-shifting and -sharing, this seemed to be effective in all health care facilities regardless of their category, although the tasks of each actor were not clearly defined. In fact, no job description was available. PMTCT interventions were offered either by a gynaecologist, a general practitioner, a midwife, a caregiver, or a psychosocial chaperone depending on their availability and planification at the level of each health care facility.

About HIV counselling and testing, the pre-screening counselling was done through educational group talks during antenatal consultations. This is confirmed by these words from a key informant during his interview: “*I attend prenatal consultations during which I conduct group counselling to promote HIV-screening”.* Nevertheless, observations figured out that the holding of this group counselling was not systemically undertaken during antenatal consultation sessions. In addition, HIV-related issues including breastfeeding of the new-born, early HIV diagnosis, and nevirapine and cotrimoxazole administration to the HIV-exposed baby were not always addressed. Furthermore, there was no prior planning or preparation of these educational talks before they were conducted. No canvas was available. In general, there was no interaction between the healthcare professional and women participating in these sessions. These talks were mainly under the responsibility of psychosocial chaperones; hence they would not always be provided if these latter were absent. No visual support dedicated to this activity was identified in the seven sites visited.

Regarding HIV testing, the proposal and completion of HIV-testing was systematic for each woman visiting the health care facility, even though the client’s consent was not systematically sought. This activity was carried-out by all healthcare professionals involved in PMTCT regardless of their qualifications. We heard from one of the psychosocial chaperones interviewed that "*Each healthcare professional working here systematically requests for HIV-testing to women received either during antenatal consultations or in the delivery room*”.

In each health care facility where they were found, psychosocial chaperones were at the forefront of care delivery to infected pregnant women and breastfeeding mothers including in the reception and orientation ward, or during counselling and therapeutic education.,. Indeed, they helped in carrying-out group counselling sessions during antenatal consultations and post-test counselling; they also provided psychosocial support for HIV-positive women and followed-up the mother–child couple including performing early HIV diagnosis of exposed infants. Although having not received any training in the clinical management of HIV-positive patients, majority of these personnel were deeply involved in ART initiation and dispensation. They were also involved in the filling of all HIV-related tools, although they had received no specific training in this regard. Precision should be made that these 2 latter activities were not part of their standard deliverables.

Concerning the provision of care and therapeutic follow-up, ART dispensation was effective in the maternal, neonatal and child health unit within five of the seven health care facilities visited. This ART dispensation was usually done during antenatal or postnatal follow-up appointments. The unit also offered support towards therapeutic adherence, early diagnosis of exposed infants and psychosocial support. However, in the two other health care facilities, women were oriented to the HIV-care unit located outside the maternal, neonatal and child health service. All participants interviewed acknowledged to be fully implicated in implementing PMTCT interventions in their respective workplace, even though majority of them had never been trained, accordingly. One of them said: “*Despite that I have not been trained for the purpose […] my responsibility is to make sure that children born from HIV-infected women end-up without getting contaminated*”.

It should be noted that identification and search of women who have not responded to their appointments or are lost to follow-up is part of the post-therapeutic follow-up. This activity was carried-out only in health care facilities where psychosocial chaperones were recruited. This was indeed part of their deliverables. They proceeded by phone calls and home visits. In this regard, they received airtime and transport costs monthly, provided by the National AIDS Control Program. However, in sites without psychosocial chaperones, there was no clear strategy in place for the active search of patients lost to follow-up. In some cases, community health care workers could be of constructive help, as attested one of our interviewees: “*When I realize that a woman is no longer attending her follow-up visits, I call for a community health care worker working in my health area, and I give him/her the patient’s contact number so that he/she can search for her in the community*”. This strategy remained however informal; it was not found in any of the health care facilities reports.

Regarding the filling of monitoring and tracking tools and registers, it was observed that follow-up registers for antenatal consultations, delivery room and laboratory were present in six of the seven sites. However, many gaps were identified including data completeness. These gaps were higher in health care facilities lacking agents in charge of filling registers who had been put at the disposal of the facility by the National AIDS Control Program. They were found in only four sites. Patients’ files were systematically used in only one facility (the regional hospital), though they were also found in 3 other ones (the district hospital and the two divisional medical centres) but inconsistently used. On the other hand, exposed new-borns’ follow-up files were present in all sites. But in majority of cases, only information pertaining to the first postnatal visit were reported. Hence, results of early HIV diagnosis were not found in these files, in all the study sites. Furthermore, registers for the monitoring and follow-up of cohorts of exposed infants between six weeks and final HIV diagnosis at 18–24 months were not found in all the study sites.

#### Training of health care professionals

Of the 16 interviewees, only the directors/managers had received a specific training on HIV-related PMTCT interventions. All other health care professionals in their facilities had received no specific training, except some coaching provided by these managers to their personnel. No mechanism of continuous training or refreshing was put in place to ensure the sustainability of knowledge and skills acquired, especially for those who had been trained. One of the managers indicated nonetheless that some refresher sessions were being conducted in his health care facility during the monthly personnel meeting, especially when one personnel had raised some difficulties faced in the course of his/her duties or activities. However, this was an internal activity and not objectivated in any of the facility reports.

#### Coordination of PMTCT services

Six of the seven study sites had a referent designated for the coordination of PMTCT services; however, we found no memo formalizing this designation. Moreover, their responsibilities were not clearly defined. The referent was supposed to coordinate counselling activities before and after testing, ART initiation and dispensation, post-therapeutic follow-up, support to adherence, psychosocial support, and follow-up of the mother–child couple. Accordingly, a respondent said: “*My role is to accompany HIV-positive women, to follow-up pregnant women during their appointment visits and during delivery at the maternity ward, and to assure early HIV diagnosis for the new-born along with monthly follow-up visits for the mother and her child*”.

#### Supervision of PMTCT interventions

None of the evaluated health care facilities had received an external supervision for the past 12 months. Nevertheless, internal supervisions were carried-out by the manager in one of the study sites. Health care professionals from this facility acknowledged positive impacts of these internal supervisions on their daily activities and performances.

### Results of the quantitative analysis

A total of 106 HIV-positive breastfeeding women were included in this study. Participants’ ages ranged between 15 and 42 years with a median of 28 years (IQR 25–32). Almost half of these women had attended at least the secondary school (50.1%), and majority of them were married (87.4%). However, less than half of these women (42.6%) had an independent source of income (Table 1). As HIV-infected patients, they had been followed-up for 5 months to 14 years, with a median duration of 3 years (IQR 2.0–5.8).

**Table 1:** Sociodemographic background of the study population

**Characteristic**		**Number (*N* = 106)**	**Percentage (%)**
**Age**	< 35	90	84.9
	≥ 35	16	15.1
**Level of education**	None	21	19.8
	Primary	32	30.1
	Secondary	48	45.3
	University	5	4.8
**Marital status**	Single/Divorced/Widow(ed)	13	12.6
	Married	81	87.4
**Any source of income**	Yes	45	42.6
	No	61	57.4

#### Knowledge on PMTCT of HIV

Sixteen questions were asked to evaluate respondents’ knowledge on PMTCT of HIV (Table 2). The number of correct answers varied from 4 to 16, with a median of 11 correct answers (IQR 8–14). For instance, women were not well informed of various advantages of ART either for them, their partner, or their new-born, including the duration of nevirapine administration to the new-born (Table 2).

**Table 2:** Respondents’ knowledge on prevention of mother-to-child transmission of HIV

Assertion	Number^*^ (*N* = 106)	Percentage (%)
HIV can be transmitted during pregnancy	76	71.7
HIV can be transmitted during childbirth	103	97.2
HIV can be transmitted during breastfeeding	101	95.3
ART is to be taken for life	100	94.3
ART helps protect my baby	55	51.9
ART helps protect my partner	47	44.3
ART helps improve my quality of life	56	52.8
ART reduces the viral load	94	88.7
ART strengthens my immunity	56	52.8
I have to practice exclusive breastfeeding for 6 months	96	90.6
Food diversification begins at 6 months	98	92.4
Breastfeeding remains possible until 12 months	24	22.6
I know that I have a follow-up visit with my newborn at 6 weeks	77	72.6
The duration of Nevirapine administration is known	49	46.2
Early HIV diagnosis of the newborn occurs between 6–8 weeks	92	86.8
Vaccination helps protect my baby	105	99.0

*ART* Antiretroviral therapy

^*^ Answered yes to the question

#### Attitudes and practices towards PMTCT of HIV

Table 3 depicts respondents’ major attitudes and practices regarding PMTCT of HIV. Only 45.3% of women had attended at least 4 antenatal consultations. Besides, only 56.6% of them never failed in taking their ART, 28.3% of them had had their CD4 count levels measured. Moreover, 19.8% of them never shared their status with their partner (Table 3). Reasons for doing so included the fear of the partner’s reaction (47.4%), not yet being ready to share the information (31.6%), keeping the status secret (15.8%), and other unspecified personal reasons (4.8%).

**Table 3:** Respondents’ attitudes and practices towards prevention of mother-to-child transmission of HIV

**Characteristic**		**Number (*N* = 106)**	**Percentage (%)**
Number of antenatal consultations performed during pregnancy	< 4	58	54.7
	≥ 4	48	45.3
During counselling, enough information was provided on HIV-screening	Yes	72	67.9
	No	34	32.1
Participated in educational sessions	Yes	94	88.7
	No	12	11.3
Has failed in taking medication	Never	60	56.6
	Rarely	31	29.2
	Often	15	14.2
CD4 count measured	Yes	30	28.3
	No	76	71.7
Has shared her status with her partner	Yes	85	80.2
	No	21	19.8

## Discussion

This mixed qualitative and quantitative analysis showed that task shifting and sharing seemed effective in all included facilities, although majority of staffs had received no adequate training on PMTCT, and their different roles and responsibilities were not clearly defined. The integration of PMTCT within maternal, neonatal, and child health services was not effective in all facilities, especially in facilities with high patients’ loads. Although HIV-screening was systematic during antenatal consultations, educational sessions and group counselling were infrequent, with no existing canvas. Pre- and post-test counselling were conducted in all facilities, but without respect to the norms and sufficient critical information sharing. ART initiation and dispensation was also effective in all facilities; however, there was insufficient follow-up of the mother–child couple, and patients lost to follow-up were not systematically identified and sought. A PMTCT referent personal had been designated in all sites, although there was no formal administrative memo and clear job description. No external supervision had been carried-out for almost a year. Moreover, more than half of HIV-positive women interviewed had a level of knowledge on PMTCT less than 80%, and their attitudes and practices were not optimal enough to guarantee a minimal risk of MTCT of HIV.

To ensure effective access to maternal and neonatal health care, especially in a context of low quantity and mostly unqualified healthcare professionals, WHO recommended task shifting and sharing [15]. Although this seemed to be effective in all study sites, it was inconsistently applied with no clear definition of each actor’s roles and responsibilities. As a consequence, optimal quality of service delivered and complete service provision cannot be guaranteed. Moreover, and despite that integration of services is part of PMTCT National Guidelines and several other international recommendations including the 2015 Dakar Appeal [7, 8, 16], this strategy remains weakly implemented, especially in facilities receiving a high number of patients. This may be explained by insufficient infrastructures or their narrowness, as well as lack of preservation of patients’ confidentiality as confirmed by a study conducted in Senegal [17]. This results in inadequacies in patients’ follow-up and thus increased numbers of lost to follow-up with consequential increased risk of MTCT of HIV. Nevertheless, psychosocial chaperones have shown to play a key role regarding HIV-infected women’s psychosocial support and identification and search of those lost to follow-up. Their presence has significantly ameliorated the quality of HIV-infected patients’ follow-up including pregnant and breastfeeding women [10]. Therefore, they should be part of the teams in charge of PMTCT programs in all health care facilities. Besides and considering that they are often assigned other important tasks such as ART dispensation and filling of monitoring tools without any training, it would be relevant to update their training modules, accordingly. In addition, they should be kept continuously trained and supervised.

This study revealed that a PMTCT referent healthcare professional is effectively designated in health care facilities to coordinate PMTCT activities. This allows a better follow-up of the implementation of PMTCT strategies within the facility, as highlighted by Kedote et al*.* in Benin [18]. Indeed, this study showed that the designation of a PMTCT referent personnel could improve service delivery and better supervision and implementation of PMTCT interventions within the facility. However, the same study showed that the lack of definition of responsibilities had a negative impact on quality of services delivered [18]. In line with these observations, we found that despite their motivation, the absence of clear job description and definition of responsibilities led to shortcomings in the application of certain management guidelines including educational talks and counselling. These activities did not respect the norms and standards upon which they should be conducted. This might result in delivering inappropriate or incomplete messages with no consequential positive improvement in women’s knowledge, attitudes, and practices towards PMTCT, ending-up in increasing the risk of getting their new-borns contaminated.

Only around 25% of health care professionals had received specific trainings on PMTCT, mainly the facility manager. Our observations are not far from that reported Fouedjio et al*.* in Yaoundé, Cameroon [19], who found that only 45.7% of the staff interviewed had received a PMTCT specific training. Obviously, this situation does not favour an optimal implementation of PMTCT activities. Accordingly, regular PMTCT trainings should be planned and healthcare professionals’ knowledge and skills should be sustained through continuous trainings, briefings, coaching, and supervisions. In addition, internal briefings and supervisions should be promoted at health care facility level.

Almost 90% of women had taken part in educational sessions, higher than what was reported by Kedote in Benin, around 50% [18]. Moreover, 80.2% of women acknowledged to have shared their status with the partner, contrasting with Hardon et al*.* results indicating that only 33% of women had shared their status with their partner, explained in majority of cases by fear of the partner’s reaction [20]. This reason was also the prevailing one in our study. More than half of women had attended at least 4 antenatal visits during their pregnancy, which corroborates the 2014 Multiple Indicators Cluster Survey results, as well as the latest DHS [5, 21]. This was nonetheless higher than findings from Diallo et al*.* in Guinea where the average number of antenatal visits conducted was 2 [22]. This unsatisfactory performance could be partly explained by insufficient women’s knowledge on the importance of antenatal care, lack of interest, stigmatization due to their HIV status, inaccessibility of the health care facility, and financial dependence to the husband/partner. This lower uptake of antenatal care constitutes a bottleneck in eliminating the MTCT of HIV. Accordingly, effective measures should be taken to increase the uptake of antenatal care among HIV-infected pregnant women. For instance, strong advocacy should be made towards administrative, traditional, and religious authorities, and local community leaders for their full engagement. In addition, male partners should be encouraged to prompt and support their wives in seeking antenatal care [23]. As clearly demonstrated, women’ health can be improved with involvement of male partners [24]. All forms of media channels should also come into play and contribute to educate these vulnerable populations.

It was found that women had good knowledge of when MTCT of HIV can occur, aligning to Harms et al. findings in Tanzania and Uganda [25]. However, according to the 2014 MICS results for the Adamawa region, 70.5, 72.4 and 71.8% of the general population of women indicated that MTCT of HIV can occur during pregnancy, delivery, and breastfeeding, respectively [21]. In Togo, Tatagan et al*.* obtained respective responses of 41.9, 46.7 and 57.6% [26]. We found that less than half of women knew the positive effects of ART therapy for them, their partners, and their new-born. Most of them were not aware of the duration of nevirapine administration. These are important areas of knowledge to be improved, for the reduction of the MTCT of HIV in our context.

This study should be interpretated in the context of some limitations. Although the objective was to evaluate PMTCT strategies across the whole country, the study was limited to one region and seven health care facilities. In addition, these sites were not chosen on a random basis as well as key informants interviewed and beneficiaries finally included, which may all together hamper the translatability of our results to the entire country. Some interviewees key demographic data were not collected (including age, sex, residence, and seniority), mainly because they were reluctant to provide these data despite having guaranteed full confidentiality, which might impact the interpretation of some of our findings. Nevertheless, we opted for a purposeful sampling taking into account the health care facility category, the patients’ load, and geographic location (urban vs rural). Besides, we used a rigorous methodology and approach combining a qualitative and quantitative analysis. Furthermore, and to the best of our knowledge, this study is one of the rare in sub-Saharan Africa that have evaluated HIV-related PMTCT intervention strategies.

## Conclusion

This mixed qualitative and quantitative analysis showed that HIV-related PMTCT strategies are well known and accepted by healthcare professionals. Majority of PMTCT interventions including integration of PMTCT within maternal, new-born and child health services, task shifting and sharing, ART initiation and dispensation, and care delivery towards voluntary HIV-screening for the mother and early HIV diagnosis for the exposed new-born are being implemented, though with some major inconsistencies. Accordingly, more emphasis should be made around reinforcing integration of services, clearly defining roles and responsibilities of healthcare professionals involved in PMTCT and organizing regular trainings, and supervisions of these personnel. Moreover, beneficiaries’ attitudes and practices regarding PMTCT are not optimal; in this regard, more advocacy and education should be made to increase them, especially antenatal consultation rates and adherence to ART. Moreover, their level of knowledge towards PMTCT interventions is suboptimal enough to guarantee a low risk of MTCT of HIV, which needs therefore to be strengthened through high quality educational sessions during visits at the health care facility level, and strong community, male partner, and media engagements.

## Supplementary Information

**Additional file 1:** **Supplementary Table 1. **Standard package of facility-based PMTCT care pre-, per-, and post-partum, and to the exposed infant. **Supplementary Table 2.** Analysis of strengths, weaknesses, opportunities and threats associated with implementation of HIV-related prevention of mother-to-child transmission strategies.

## Data Availability

The datasets used and/or analyzed during the current study are available from the corresponding author on reasonable request.

## Abbreviations

AIDS: Acquired immunodeficiency syndrome
ART: Antiretroviral therapy
DHS: Demographic and Health Survey
HIV: Human immunodeficiency virus
PMTCT: Prevention of mother-to-child transmission
WHO: World Health Organization
