# Supplementary material for: Evaluation of strategies for the prevention of mother-to-child transmission of HIV in Cameroon: a mixed qualitative and quantitative cross-sectional analysis from the Adamawa region of Cameroon
Source: BMC Public Health. 2022 Dec 31;22:2459. doi: 10.1186/s12889-022-14871-6 (PMC9805060; doi:10.1186/s12889-022-14871-6)
Supplement: Supplementary file 1 — Additional file 1: Supplementary Table 1. Standard package of facility-based PMTCT care pre-, per-, and post-partum, and to the exposed infant. Supplementary Table 2. Analysis of strengths, weaknesses, opportunities and threats associated with implementation of HIV-related prevention of mother-to-child transmission strategies. [file 12889_2022_14871_MOESM1_ESM.docx]

**Supplementary Table 1:** Standard package of facility-based PMTCT care pre-, per-, and post-partum, and to the exposed infant [8]

| **Package of services for pregnant women during antenatal consultations (ANC)** | | | | | | | | | | | | | | | | | | |  |  |
| --- | --- | --- | --- | --- | --- | --- | --- | --- | --- | --- | --- | --- | --- | --- | --- | --- | --- | --- | --- | --- |
| **Interventions** | | **ANC1** | | | **ANC2** | | | | | | **ANC3** | | | | | **ANC4** | | |  |  |
| **Period of visits** | | 1st trimester: anytime before **16th week** | | | 2nd trimester : **24-28 weeks** | | | | | | 3rd trimester : **32-36 weeks** | | | | | 4th trimester : **after 36 weeks** | | |  |  |
| **Clinical examination** | | X | | | X | | | | | | X | | | | | X | | |  |  |
| **Pelvic evaluation** | |  | | |  | | | | | | X | | | | | X | | |  |  |
| **Laboratory tests** | | | | | | | | | | | | | | | | | | |  |  |
| **HIV test** | | Pregnant women and her patner if HIV status is unknow | | | Pregnant women and her patner are not yet tested | | | | | | Repeat HIV test if the first one was negative | | | | | | | |  |  |
| **Blood group, Rh** | | Determine the ABO group and the Rh factor from the first consultation | | | | | | | | | | | | | | | | |  |  |
| **FBC, Hb** | | - Do FBC if the woman shows signs of anemia (pallor) - **If Hb <11.5g/l**, if the pregnant woman is anemic: double the dose of iron and folic acid and give nutritional advice | | | | | | | | | | | | | | | | |  |  |
| **Transaminases** | | X | | |  | | | | | | X | | | | |  | | |  |  |
| **Fasting Blood Sugar** | | X | | |  | | | | | | X | | | | |  | | |  |  |
| **Urines** | | Check for sugar (diabetes check) and albumin at each visit | | | | | | | | | | | | | | | | |  |  |
| **Syphilis** | | Do TPHA/VDRL | | | Do TPHA/VDRL if not done previously | | | | | | | | | | | | | |  |  |
| **Viral hepatitis** | | Do HBs Ag test systematically to all pregnant women during the 1st ANC (or 1^st^ contact with the ANC service) | | | | | | | | | | | | | | | | |  |  |
| **CD4 count** | | X | | |  | | | | | | X | | | | |  | | |  |  |
| **Viral Load (VL)** | | VL if already on antiretroviral therapy (ART) with VL for more than one month | | | VL at 3 months after ART initiation and then 3 months after last VL | | | | | | | | | | | | | |  |  |
| **Treatment and Prophylaxis** | | | | | | | | | | | | | | | | | | |  |  |
| **Vaccination** | | Give the first dose of tetanus vaccine if not vaccinated | | | Give the 2^nd^ dose of tetanus vaccine (at least 4 weeks before the first dose) if applicable | | | | | | Give a dose of tetanus vaccine if needed (if she has not yet received 2 doses since the start of pregnancy) | | | | | | | |  |  |
| **Supplementation** | | Iron/Folic acid: 1tablet of iron (200mg) and 1 tablet of folic acid (5mg) daily. In case of anaemia, double the doses | | | | | | | | | | | | | | | | |  |  |
| **Mebendazole** | | Do not give during the 1^st^ trimester | | | Give 1 tablet of 500 mg at the second or the third trimester | | | | | | | | | | | | | |  |  |
| **Intermittent preventive treatment (IPT) of malaria** | | Do not administer before 16th week of pregnancy, advise for long-lasting insecticide-treated bed nets (LLITN) | | | Give SP 500mg/25mg.  If HIV+, give CTX instead of IPT, advise for LLITN | | | | | | | | | | | advise LLITN | | |  |  |
| **Cotrimoxazole (CTX)** | | Give CTX at each visit if HIV+ | | | | | | | | | | | | | | | | |  |  |
| **ART** | | Give ART as soon as possible after diagnosis regardless of pregnancy term, CD4 count or clinical stage | | | | | | | | | | | | | | | | |  |  |
| **Support to ART adherence** | | At each visit | | | | | | | | | | | | | | | | |  |  |
| **Breastfeeding advice** | |  | | |  | | | | | | X | | | | | X | | |  |  |
| **Preparation to childbirth** | |  | | |  | | | | | | X | | | | | X | | |  |  |
|  | | | | | | | | | | | | | | | | | | |  |  |
| **Package of services for HIV-positive women during post-partum** | | | | | | | | | | | | | | | | | | |  |  |
| **Period of visits** | | | During the week following delivery | | | | | | From the 6^th^ week to the 6^th^ month | | | | | From 6^th^ to 24th month | | | | |  |  |
| **Follow-up** | | | 6 days after delivery | | | | | | Once monthly | | | | | Every 3 months | | | | |  |  |
|  |  |  | - Screen concurrent conditions of the mother and treat them - Provide health education on maternal and child nutrition, vaccinations, family planning, hygiene, and prevention of sexually transmissible infections - Promote dialogue with the partner within the couple and responsible parenthood - Assessment of adherence to ART and support needed | | | | | | | | | | | | | | | |  |  |
| **Family planning** | | | - Provide counseling on family planning and available methods and start contraception if needed | | | | | | | | | | | | | | | |  |  |
| **Physical examination** | | | - Examination of the abdomen, vagina, cervix - Examine the baby's anterior fontanel and healing of the umbilicus | | | | | | - Examination of the abdomen, vagina, cervix - Do the vaginal Pap smear | | | | | | | | | |  |  |
| **Alarming signs** | | | - Inform the woman of alarming signs to monitor during post-partum, and encourage her to immediately return back to the health care facility accordingly - **Mother**: vaginal bleeding, seizures, difficulty breathing, fatigue, fever, abdominal pains, pallor, oedema, vaginal discharge - **Baby**: red or purulent cord, refusal to breastfeed, swollen eyes, sticky or draining pus, baby cold to the touch in hot weather or baby hot when undressed, difficulty breathing, lethargy, pale or yellow eyes, repeated vomiting, seizures | | | | | | | | | | | | | | | |  |  |
| **Nutritional evaluation** | | | Measure the infant’s weight, height and head circumference, and compare with standard values for his/her age; in case of malnutrition, provide nutritional support and/or refer the case | | | | | | | | | | | | | | | |  |  |
| **Screening for Tuberculosis** | | | Systematic | | | | | | | | | | | | | | | |  |  |
| **Biological exams** | | | | | | | | | | | | | | | | | | |  |  |
| **HIV testing** | | | Testing to be offered to the mother and her partner if unknown serology or negative test dating of more than 3 months | | | | | | | | | | | | | | | |  |  |
| **Early HIV diagnosis for the exposed infant** | | | - Perform HIV testing of the exposed new-born through DBS/PCR or POC/EID at 6-8 weeks - Continue the diagnosis procedure according to the national algorithm - For every child found positive to HIV, initiate ART as soon as possible, and assure good adherence and retention on care | | | | | | | | | | | | | | | |  |  |
| **Viral load** | | | To be performed every three months during breastfeeding | | | | | | | | | | | | | | | |  |  |
| **Treatment and prophylaxis** | | | | | | | | | | | | | | | | | | |  |  |
| **Iron/Folic acid** | | | Give 1 tablet of iron (200mg) + 1 tablet of folic acid (5mg) per day during 3 months; in case of anaemia, double the doses | | | | | | | | | | | | | | | |  |  |
| **Mebendazole** | | | If the patient had not received during pregnancy, give Mebendazole (single dose) at delivery and subsequently every 6 months | | | | | | | | | | | | | | | |  |  |
| **Intermittent preventive treatment of malaria** | | | Give advice to the mother regarding the use of long-lasting insecticide-treated bed nets; if necessary, provide if available | | | | | | | | | | | | | | | |  |  |
| **Cotrimoxazole** | | | Provide Cotrimoxazole to HIV-infected women | | | | | | - Administer Cotrimoxazole to HIV-infected women - Administer Cotrimoxazole to the exposed infant from 6 weeks until confirmation of non-contamination (negative status) | | | | | | | | | |  |  |
| **ART** | | | Initiate or continue administering ART to the HIV-infected mother, and initiate Nevirapine to be given to the exposed new-born within the first 72 hours after delivery until 6 weeks | | | | | | | | | | | | | | | |  |  |
|  | | | | | | | | | | | | | | | | | | |  |  |
| **Follow-up of exposed infants born from HIV-infected mothers** | | | | | | | | | | | | | | | | | | | | |
|  | **At birth** | | | **At six weeks (W6)** | | **From week 6 to month 6 (monthly visits)** | | | | | | **From month 6 to month 18-24 (quarterly visits)** | | | | | | | | |
|  |  | | | - Evaluate adherence to prophylactic ART - Diagnose and treat concurrent infections - Perform HIV testing through PCR - Link positive cases to the ART dispensation service | | At each visit:   - Screen for concurrent pathologies and treat them accordingly - Perform HIV testing through PCR - Link positive cases to the ART dispensation service | | | | | | At each visit :   - Screen for concurrent pathologies and treat them accordingly - HIV serology at 9 months and confirmation by PCR if positive serology - HIV serology at 18 months or 6 weeks after termination of breastfeeding - Link positive cases to the ART dispensation service | | | | | | | | |
|  |  | | |  | | **W10** | **W14** | **M5** | | **M6** | | **M9** | **M12** | | **M15** | | **M18** | **M21** | | **M24** |
| **Prophylactic ART** | **X** | | |  | |  |  |  | |  | |  |  | |  | |  |  | |  |
| **Psychosocial support** | **X** | | | **X** | | **X** | **X** | **X** | | **X** | | **X** | **X** | | **X** | | **X** | **X** | | **X** |
| **Growth follow-up** | **X** | | | **X** | | **X** | **X** | **X** | | **X** | | **X** | **X** | | **X** | | **X** | **X** | | **X** |
| **Surveillance of psychomotor development** | **X** | | | **X** | | **X** | **X** | **X** | | **X** | | **X** | **X** | | **X** | | **X** | **X** | | **X** |
| **Full clinical examination** | **X** | | | **X** | | **X** | **X** | **X** | | **X** | | **X** | **X** | | **X** | | **X** | **X** | | **X** |
| **Feeding counselling** | **X** | | | **X** | | **X** | **X** | **X** | | **X** | | **X** | **X** | | **X** | | **X** | **X** | | **X** |
| **Vaccination** | **X** | | | **X** | | **X** | **X** |  | |  | | **X** |  | |  | |  |  | |  |
| **Cotrimoxazole initiation** |  | | | Begin cotrimoxazole at 6 weeks and continue until confirmation of negative status | | | | | | | | | | | | | | | | |
| **HIV diagnosis of the exposed infant** |  | | | **PCR** | | | | | | | | **PCR at 9 months** | | | | | **HIV serology** | | | |

**Supplementary Table 2: Analysis of strengths, weaknesses, opportunities and threats associated with implementation of HIV-related prevention of mother-to-child transmission strategies**

| **Thematic** | **Component** | **Strengths** | **Weaknesses** | **Opportunities** | **Threats** |
| --- | --- | --- | --- | --- | --- |
| **Organization of services, service delivery and compliance with management** **guidelines** | **Integration of services** | Implemented in majority of HCF | Not implemented in HCF with high patient loads  Loss of patients in the care circuit | Existence of the PBF project which buys indicators for HIV-screening, ART initiation and follow-up of exposed infants, upon compliance to PMTCT national guidelines and proper use of monitoring tools including registers and patients’ files, and active search of patients loss to follow-up |  |
|  | **Task shifting and sharing** | Seeming effective in all HCF  Majority of staff highly motivated | No description of every staff’s tasks and responsibilities |  |  |
|  | **HIV counselling and testing** | Educational talks and counselling conducted in all HCF  Presence of trained psychosocial chaperones who have this activity as part of their deliverables | No harmonization of educational sessions canvas  Insufficient information sharing during counselling |  |  |
|  | **Care delivery in therapeutic follow-up** | ART dispensation, and follow-up of the mother-child couple in MNCH services in majority of HCF  Identification and search for lost to follow-up by psychosocial chaperones | No integration of PMTCT within MNCH services in some HCF  Complexity of the care circuit in HCF where there is no integration of services |  |  |
|  | **Filling of monitoring tools** | Presence of harmonized registers, patients and exposed infants’ files in majority of HCF  Presence of agents responsible for the filling of registers in some HCF | Insufficiencies in the utilization of monitoring tools  Missing data due to lack of knowledge on how to fill these tools |  |  |
| **Training of health care professionals on PMTCT of HIV** |  | Trained HCF directors/managers  Staff briefings regularly conducted in some HCF | Lack of trained health care professionals who are dedicated to PMTCT  No mechanism put in place for the sustainability of acquired training skills and knowledge | Possibility of using PBF-generated funds to organize or support the training of health care professionals involved in PMTCT | Insecurity in some areas resulting in significant mobility/instability of trained personnel |
| **Coordination** |  | Designation of a PMTCT focal point in all HCF | Absence of a clear job description of the PMTCT focal point |  |  |
| **Supervision** |  | Internal supervisions conducted in some HCF by the manager | No external supervision undertaken in any of the HCF, for more than 12 months  No compliance to the national PMTCT management guidelines | Possibility of using PBF- generated funds to organize external supervisions by the health district and/or regional delegation |  |

HCF = health care facility; MNCH = maternal, neonatal and child health ; PBF = performance-based financing; PMTCT = prevention of mother-to-child transmission
